# Novel Findings on CCR1 Receptor in CNS Disorders: A Pathogenic Marker Useful in Controlling Neuroimmune and Neuroinflammatory Mechanisms in Parkinson’s Disease

**DOI:** 10.3390/ijms25084337

**Published:** 2024-04-14

**Authors:** Alberto Repici, Anna Paola Capra, Ahmed Hasan, Maria Bulzomì, Michela Campolo, Irene Paterniti, Emanuela Esposito, Alessio Ardizzone

**Affiliations:** 1 Department of Chemical, Biological, Pharmaceutical and Environmental Sciences, University of Messina, Viale Ferdinando Stagno D’Alcontres, 31, 98166 Messina, Italy; alberto.repici@unime.it (A.R.); annapaola.capra@unime.it (A.P.C.); ahmed.hasan@unicam.it (A.H.); maria.bulzomi@unime.it (M.B.); campolom@unime.it (M.C.); ipaterniti@unime.it (I.P.); aleardizzone@unime.it (A.A.); 2 School of Advanced Studies, Center of Neuroscience, University of Camerino, 62032 Camerino, Italy

**Keywords:** Parkinson’s disease (PD), Central Nervous System (CNS), immune-inflammatory pathways, chemokines, CCR1 receptor, BX471

## Abstract

Parkinson’s disease (PD) is recognized as the second most common neurodegenerative disease worldwide. Even if PD etiopathogenesis is not yet fully understood, in recent years, it has been advanced that a chronic state of inflammation could play a decisive role in the development of this pathology, establishing the close link between PD and neuroinflammation. In the broad panorama of inflammation and its several signaling pathways, the C-C chemokine receptor type 1 (CCR1) could play a key pathogenic role in PD progression, and could constitute a valuable target for the development of innovative anti-PD therapies. In this study, we probed the neuroprotective properties of the CCR1 antagonist BX471 compound in a mouse model of MPTP-induced nigrostriatal degeneration. BX471 treatments were performed intraperitoneally at a dose of 3 mg/kg, 10 mg/kg, and 30 mg/kg, starting 24 h after the last injection of MPTP and continuing for 7 days. From our data, BX471 treatment strongly blocked CCR1 and, as a result, decreased PD features, also reducing the neuroinflammatory state by regulating glial activation, NF-κB pathway, proinflammatory enzymes, and cytokines overexpression. Moreover, we showed that BX471’s antagonistic action on CCR1 reduced the infiltration of immune cells, including mast cells and lymphocyte T activation. In addition, biochemical analyses carried out on serum revealed a considerable increase in circulating levels of CCR1 following MPTP-induced PD. In light of these findings, CCR1 could represent a useful pathological marker of PD, and its targeting could be a worthy candidate for the future development of new immunotherapies against PD.

## 1. Introduction

Parkinson’s disease (PD) is one of the most common neurodegenerative diseases in the wider context of Central Nervous System (CNS) disorders [1], and it was recognized as the second most afflicting neurocognitive condition after Alzheimer’s Disease (AD) [2].

In particular, PD affects about 0.5–1% of the world’s population aged between 65 and 69 years, increasing to up to 1–3% of people who are 80 or older [3]. The typical motor symptoms that characterize PD are rigidity, tremor, bradykinesia, and postural instability which is strongly correlated with the disease’s progression [4]. Moreover, the increasing deterioration of different neurotransmitters leads to a wide variety of non-motor symptoms such as hallucinations, dementia, and difficulties in memory or spatial memory [5]. These symptoms largely affect cognitive domains in PD patients, constituting an important source of patient disability and caregiver burden [6].

Thus, it is clear that due to all the above-mentioned symptoms, PD sufferers have extreme debilitation, which has a very negative impact on the quality of life and daily activities.

It has been widely established that the trigger of symptoms is the gradual death of dopaminergic neurons in the substantia nigra as well as an extensive accumulation of α-synuclein, which aggregate in foreign bodies known as “Lewy bodies” [7].

As a multifactorial disorder, PD involves the interplay of aging, genetics, and environmental factors [8]. In this regard, PD was strongly associated with the mutation of more than 100 gens that have been identified till now like *LRRK2*, *PARK7*, *PINK1*, *PRKN*, etc. [9].

For instance, Gaucher Disease (GD), an inherited lysosomal storage disorder caused by biallelic mutations in the *GBA1* gene that encodes the glucocerebrosidase enzyme, seems to increase the risk of developing PD compared to the general population [10].

Moreover, the pathophysiology of PD appears to be closely related to chronic neuroinflammation, which is one of the primary hallmarks of PD pathophysiology. According to post-mortem examinations of PD patients’ bodies and research conducted on experimental animals, the PD brain is frequently found to have activated glial cells and elevated amounts of pro-inflammatory factors. Activated microglia and astrocytes emit pro-inflammatory cytokines on a chronic basis, which exacerbates the degeneration of dopaminergic neurons in SNC [11].

Indeed, in recent decades, the hypothesis that PD and inflammation are likely linked has been strengthened, laying the foundation for the development of new targeted treatments focusing on neuroinflammatory signaling pathways underlining neurodegeneration in PD [12].

Neuroinflammation is a highly regulated and complex process, and several markers are strictly involved in PD. This particular release of cytokines affects normal neuronal and glial activity in the brain, creating a unique network. Tumor necrosis factor (TNF)-α, transforming growth factor (TGF)-β, Interleukin (IL)-1β, and IL-6 are produced by microglia themselves. Thereby, different cytokines, including TNF-α and IL-6, are considered as potential biomarkers for PD [13]. Furthermore, one important element for PD’s start and development is IL-1β [14].

While α-synuclein is actively cleared from susceptible neurons by astrocytes in PD, the buildup of α-synuclein in their cytoplasm eventually leads to a pro-inflammatory response, adds to their defective phenotype, and propagates the disease [15].

Chemokines, a vast class of low molecular weight proteins essential for controlling the displacement of leukocytes and other cells of the immune system during the inflammation process, act through the binding of specific receptors which, once activated, trigger the amplification of cascade proinflammatory reactions [16].

In this study, our attention focused on the C-C chemokine receptor type 1 (CCR1), a specific protein belonging to the class of G protein-coupled receptors, which is a pivotal checkpoint for the modulation of immune-inflammatory mechanisms [17].

The CCR1 receptor has several endogenous ligands, among others, fall into this category CCL3 (known as MIP-1 alpha) and CCL5 (known as RANTES) [18].

CCR1’s complicated activity plays a major role in several pathways that, when altered, result in neuroinflammation and neurodegeneration. Although CCR1 and its associated components can have a variety of consequences on the nervous system, its proinflammatory characteristics were first believed to have a significant role in the emergence of CNS diseases [19].

Hence, the blocking of this protein through specific antagonists could have a positive effect on human pathologies, including CNS disorders like PD. For these reasons, it synucleinthesized the R-N-[5-chloro-2-[2-[4-[(4-fluorophenyl)methyl]-2-methyl-1-piperazinyl]-2-oxoethoxy]phenyl]urea hydrochloric acid salt (BX471), a non-peptide antagonist of CCR1 that is biologically active, strong, and selective for CCR1 and its isoforms. BX471 exhibited remarkable pharmacokinetic properties while not showing any toxicity, hemodynamics, or harmful effects on the CNS [20].

After the BX471 discovery, several preclinical studies were performed to evaluate its biological properties, highlighting that this chemical compound modulated inflammation and immune-cell infiltration in experimental mouse models of renal fibrosis [21], asthma [22], and allergic rhinitis [23].

Furthermore, the therapeutic effect of BX471 also resulted in a considerable modulation of mast cells activity and CD4^+^/CD8^+^ lymphocytes thanks to CCR1 antagonism.

So, it can be hypothesized that the regulation of the complex network involving the inflammatory pathways and related immune response activity could be also used to counteract neurodegenerative conditions such as PD. In light of this, targeted therapy against the CCR1 receptor may offer a promising therapeutic substitute to the existing standard of care for patients suffering from PD, also considering the recent involvement of α-synuclein-based immunotherapy and other immunotherapeutic interventions in clinical trials.

Therefore, by using MPTP to induce nigrostriatal degeneration in mice, this study aimed to offer a novel perspective on the role of CCR1 as a useful pathogenic marker and target in PD.

Additionally, we also assessed the therapeutic potential of BX471 in mitigating immune-inflammatory mechanisms that have been altered by the establishment of neurodegeneration.

## 2. Results

### 2.1. Blocking of CCR1 Exerted by BX471 Reduced the Behavioral Deficits Caused by MPTP-Induced Nigrostriatal Degeneration

**Figure 1:**
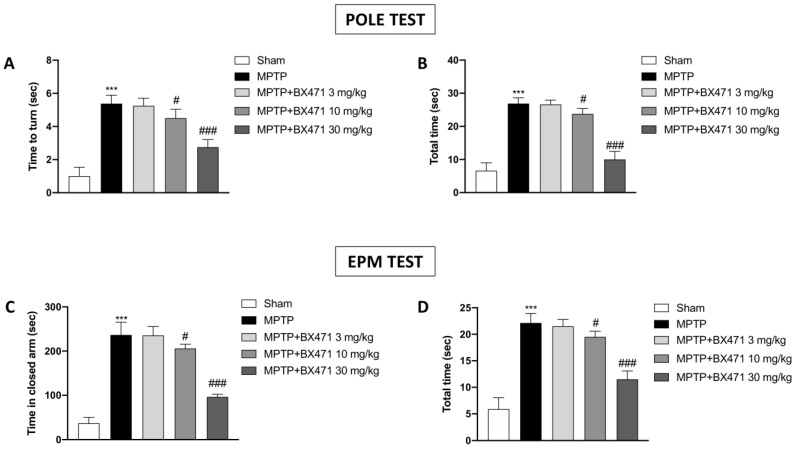
Evaluation of BX471 on behavioral disorders induced by MPTP. Graphs related to Pole Test highlighting “Time to turn” (**A**) and the “Total time” (**B**) are shown. EPM test was also performed, “Time in closed arms” (**C**), and the “Total time” (**D**) was assessed. In every experimental group, the number of mice was *n* = 8. Values are means  ±  SD. One-Way ANOVA test. *** *p*  <  0.001 vs. Sham; # *p*  <  0.05 vs. MPTP; ### *p*  <  0.001 vs. MPTP.

Patients with PD often experience emotional changes and motor deficits. Therefore, we first evaluated if the CRR1 receptor block improved mice. In particular, the pole test was utilized to assess the bradykinesia and motor change brought on by MPTP intoxication. Behavioral outcomes showed that MPTP-injected mice had longer “Time to turn” and “Total time” than the Sham group (Figure 1A,B). While CCR1 antagonistic treatment by using BX471 at the dose of 30 mg/kg resulted in a significant reduction in “Time to turn” and “Total time,” indicating a good improvement from bradykinesia (Figure 1A,B). Likewise, the group treated with BX471 10 mg/kg exposed a lesser but noteworthy decrease in motor impairments (Figure 1A,B).

Moreover, we employed one of the most common anxiety-like behaviors, such as EPM, to evaluate anxiety. When compared to the Sham group, during EPM, we observed that MPTP-intoxicated mice spent a higher proportion of their time in the closed arm and in the center (Figure 1C,D, respectively). Rather, treated mice with BX471 10 mg/kg, and particularly at the dose of 30 mg/kg, significantly decreased the amount of time spent in the closed arms (Figure 1C,D).

On the other hand, both behavioral tests did not demonstrate a significant improvement following BX471 3 mg/kg administration (Figure 1A–D).

### 2.2. Antagonism of CCR1 by BX471 Inhibited the Loss of TH^+^ Neurons following MPTP-Induced Nigrostriatal Degeneration

Since TH is a crucial enzyme in the production of dopamine, we used IHC to examine whether BX471’s suppression of CCR1 could protect striatal dopaminergic neurons from MPTP-induced loss.

**Figure 2:**
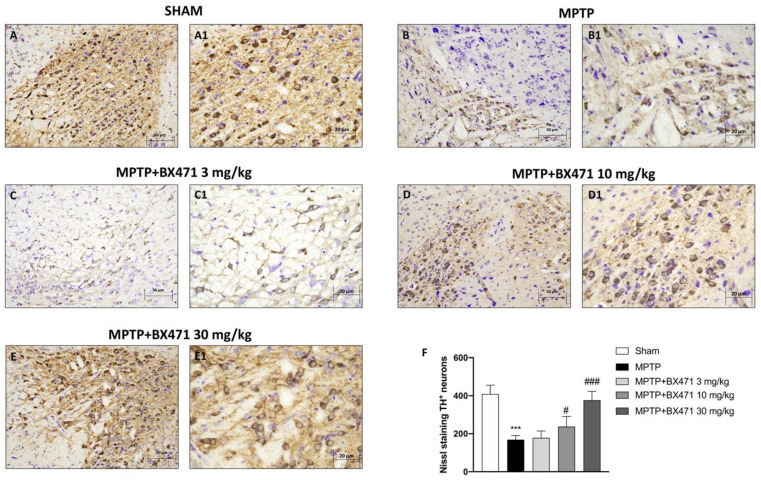
Evaluation of BX471 on TH expression. Representative images of brain sections stained with TH and counterstained with Cresyl violet for each experimental group, Sham (**A**,**A1**), MPTP (**B**,**B1**), MPTP + BX471 3 mg/kg (**C**,**C1**), MPTP + BX471 10 mg/kg (**D**,**D1**), MPTP + BX471 30 mg/kg (**E**,**E1**). Nissl score (**F**). In every experimental group, the number of mice was *n* = 8. Figures are shown at 20× and 40× magnification. Values are means  ±  SD. One-Way ANOVA test. *** *p*  <  0.001 vs. Sham; # *p*  <  0.05 vs. MPTP; ### *p*  <  0.001 vs. MPTP.

We found that the MPTP-injected animals had significantly fewer TH^+^ neurons (Figure 2B,B1, score Figure 2F) than the Sham group (Figure 2A,A1, score Figure 2F). When BX471 was administered at a dosage of 3 mg/kg, the loss of TH-positive neurons caused by MPTP was not significantly reduced (Figure 2C,C1, score Figure 2F).

In contrast, compared to MPTP-injected midbrain sections, treatment with BX471 at a dose of 10 mg/kg (Figure 2D,D1, score Figure 2F) and mainly at 30 mg/kg (Figure 2E,E1, score Figure 2F) notably preserved the number of TH^+^ neurons.

### 2.3. Downregulation of CCR1 by BX471 Protected against MPTP Neurotoxicity-Induced DAT Depletion

**Figure 3:**
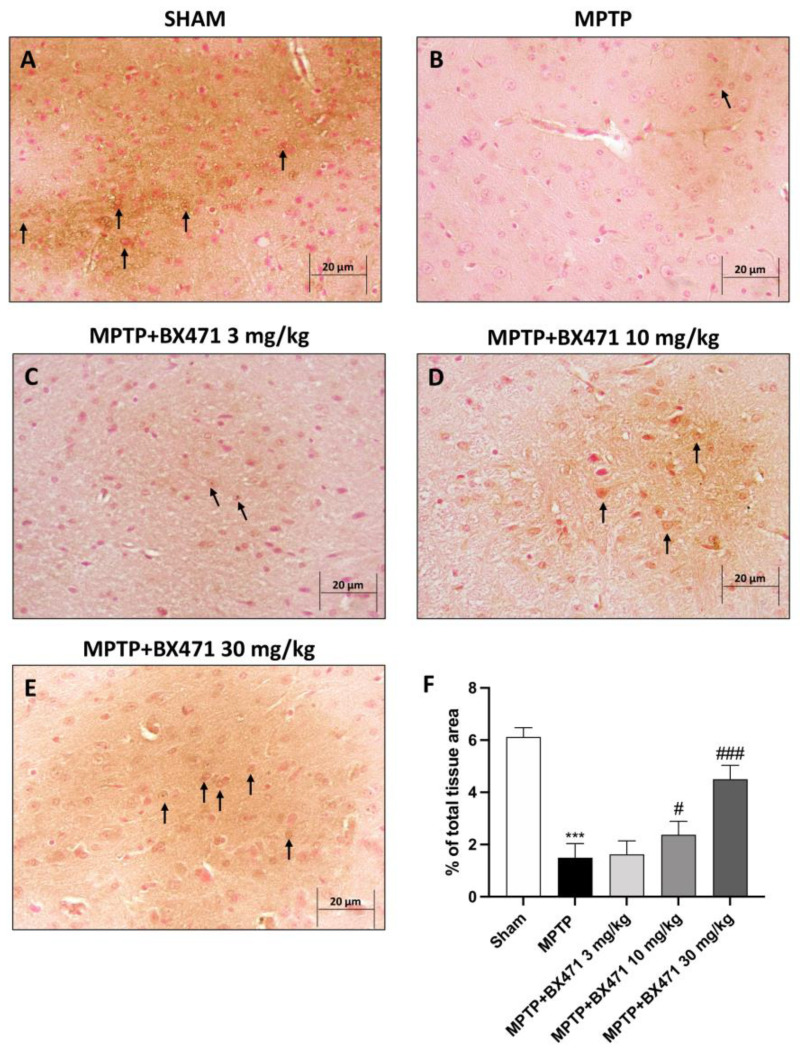
Evaluation of BX471 treatment on DAT expression. Representative images of DAT evaluations for the following experimental groups: Sham (**A**), MPTP (**B**), MPTP + BX471 3 mg/kg (**C**), MPTP + BX471 10 mg/kg (**D**), MPTP + BX471 30 mg/kg (**E**). IHC score (**F**). In every experimental group, the number of mice was *n* = 8. Black arrows indicate positive cells. Figures are shown at 40× magnification. Values are means  ±  SD. One-Way ANOVA test. *** *p*  <  0.001 vs. Sham; # *p*  <  0.05 vs. MPTP; ### *p*  <  0.001 vs. MPTP.

We used immunohistochemical staining to assess DAT expression in the substantia nigra to further explore the protective effects of this CCR1 blocker on the dopamine system. When comparing the MPTP-injected mice to the Sham group (Figure 3A, score Figure 3F), the obtained findings showed a substantial reduction of DAT immunoreactivity (Figure 3B, score Figure 3F). In contrast, recovery of DAT levels was considerable following daily administrations of BX471 10 mg/kg (Figure 3D, score Figure 3F) and more successfully at a dose of 30 mg/kg (Figure 3E, score Figure 3F) in respect to the MPTP + vehicle group. On the other hand, DAT expression was not substantially improved by BX471 at the lowest dose (3 mg/kg) (Figure 3C, score Figure 3F).

### 2.4. BX471 Counteracted α-Synuclein Accumulation Provoked by MPTP-Induced Nigrostriatal Degeneration

To ascertain if CCR1-blocking BX471 can reverse the neurodegenerative process, we sought to assess the expression of α-synuclein, as this protein accumulation in the substantia nigra is a PD crucial marker.

**Figure 4:**
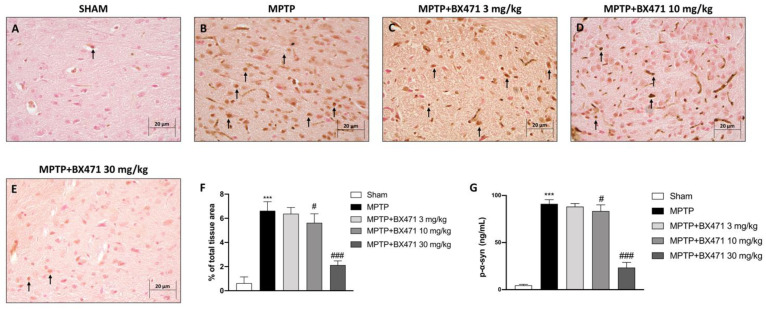
BX471 administration decreased aggregation of α-synuclein. Representative images of α-synuclein immunohistochemical staining for the following experimental groups: Sham (**A**), MPTP (**B**), MPTP + BX471 3 mg/kg (**C**), MPTP + BX471 10 mg/kg (**D**), MPTP + BX471 30 mg/kg (**E**). IHC score (**F**). ELISA kit for p-α-synuclein was also performed (**G**). In every experimental group, the number of mice was *n* = 8. Black arrows indicate positive cells. Figures are shown at 40× magnification. Values are means  ±  SD. One-Way ANOVA test. *** *p*  <  0.001 vs. Sham; # *p*  <  0.05 vs. MPTP; ### *p*  <  0.001 vs. MPTP.

In comparison to the baseline levels of α-synuclein displayed by the Sham group (Figure 4A, score Figure 4F), immunohistochemical examination of midbrain sections in MPTP-injured animals revealed a considerable buildup of α-synuclein (Figure 4B, score Figure 4F).

In addition, our findings demonstrated that BX471 10 mg/kg effectively counteracted synuclein aggregates induced by MPTP injection (Figure 4D, score Figure 4F). A higher reduction in α-synuclein deposition was detected when BX471 dosage was increased to 30 mg/kg (Figure 4E, score Figure 4F). BX471 3 mg/kg did not show any evident efficacy in decreasing α-synuclein immunoreactivity (Figure 4C, score Figure 4F).

Moreover, we also examined the p-α-synuclein form as a peculiar protein linked to PD pathophysiology to fully elucidate the neuroprotective impact of the CCR1 blocker BX471 against α-synuclein accumulation.

Elevated p-α-synuclein levels were found in the MPTP group compared to the Sham group (Figure 4G). BX471, in a dose-dependent manner, decreased p-α-synuclein concentrations, thus resulting in it being particularly effective at the dose of 30 mg/kg (Figure 4G).

Considering these preliminary results in which only the highest doses of BX471 were the most effective in counteracting PD hallmarks, reaching statistical significance, we proceeded with our analyses excluding BX471 3 mg/kg.

### 2.5. BX471 Decreased Reactive Astroglia and Microglia following MPTP-Induced Nigrostriatal Degeneration

In PD, neuroinflammation and the death of dopaminergic neurons are also caused by two pro-inflammatory mechanisms: the activation of reactive astrocytes, and microglia. Hence, we investigated GFAP and IBA-1 as recognized indicators of microgliosis and astrogliosis, respectively, by using immunofluorescence analysis.

**Figure 5:**
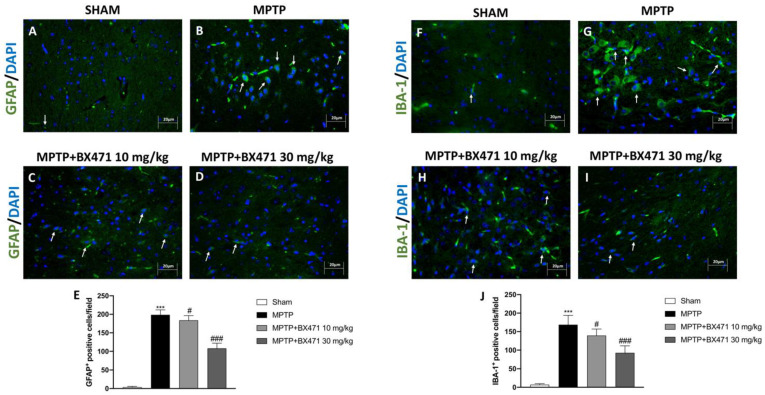
Evaluation of BX471 on GFAP and IBA-1 expression. Representative images of the immunofluorescence analysis for GFAP and IBA-1 markers, Sham (**A** and **F**, respectively), MPTP (**B** and **G**, respectively), MPTP + BX471 10 mg/kg (**C** and **H**, respectively), MPTP + BX471 30 mg/kg (**D** and **I**, respectively). Scores are indicated (**E**,**J**). In every experimental group, the number of mice was *n* = 8. White arrows indicate positive cells. Figures are shown at 40× magnification. Values are means  ±  SD. One-Way ANOVA test. *** *p*  <  0.001 vs. Sham; # *p*  <  0.05 vs. MPTP; ### *p*  <  0.001 vs. MPTP.

Our results showed a substantial increase in both IBA-1^+^ and GFAP^+^ cells following MPTP injection (Figure 5B and Figure 5G, respectively, score Figure 5E and Figure 5J) compared to the Sham animals (Figure 5A and Figure 5F, respectively, score Figure 5E and Figure 5J). Otherwise, the administration of BX471 dose-dependently decreased astrogliosis and microgliosis induced by MPTP intoxication (Figure 5C and Figure 5H, respectively, Figure 5D and Figure 5I, respectively, score Figure 5E and Figure 5J).

### 2.6. BX471 Restrained NF-kB Translocation and Reduced Pro-Inflammatory Mediators following MPTP-Induced Nigrostriatal Degeneration

The NF-κB protein functions as a “master switch” for the production of inflammatory genes, and it regulates the inflammatory mediators that play a crucial role in PD-related chronic inflammation and neuronal death. Thus, we used Western Blot analysis to assess the NF-κB pathway and the expression of pro-inflammatory mediators.

**Figure 6:**
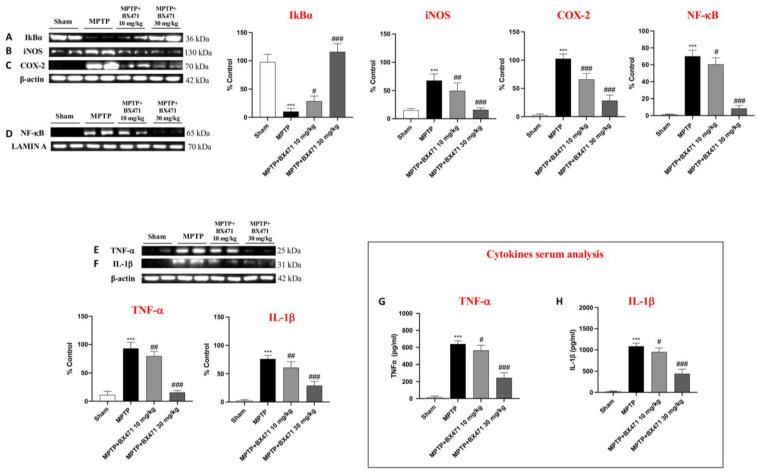
BX471 administration reduced neuroinflammation caused by MPTP. Representative blots of IkBa (**A**), iNOS (**B**), COX-2 (**C**), NF-kB (**D**), TNF-α (**E**), and IL-1β (**F**). Serum analysis of TNF-α (**G**) and IL-1β (**H**). In every experimental group, the number of mice was *n* = 8. Values are means  ±  SD. One-Way ANOVA test. *** *p*  <  0.001 vs. Sham; # *p*  <  0.05 vs. MPTP; ## *p*  <  0.01 vs. MPTP; ### *p*  <  0.001 vs. MPTP.

In the MPTP group, we observed a significant reduction in IkBα levels in contrast to the Sham group, where baseline expression of this cytosolic protein was observed (Figure 6A). However, BX471 treatment considerably restored IκBα levels, notably at the dosage of 30 mg/kg (Figure 6A). At the same time, we found that MPTP-intoxicated mice showed an increase in the nuclear translocation of NF-κB when compared to the Sham group (Figure 6B). The administration of BX471, in a dose dependent way, significantly decreased this rise (Figure 6B).

As a result of the NF-κB modulation exerted by BX471, we also identified a reduction of pro-inflammatory enzymes and cytokines such as iNOS, COX-2, TNF-α, and IL-β if compared to the MPTP + vehicle group (Figure 6C–F). Analysis on serum samples confirmed the ability of BX471 in decreasing the cytokines’ circulating levels (Figure 6G,H).

### 2.7. BX471 Antagonized the CCR1 Receptor and Moderated Immune-Inflammatory Mediators after MPTP-Induced Nigrostriatal Degeneration

The results of gene expression analysis showed a distinct PD-specific gene profile that included transcriptome indicators for oxidative stress, inflammation, mitochondrial autophagy, and, most importantly, chemokine signaling through CCR1.

Thus, to confirm that previous results were a consequence of CCR1 downregulation exerted by BX471, we evaluated specific markers of this cascade signaling pathway in the midbrain using Western Blot analysis.

**Figure 7:**
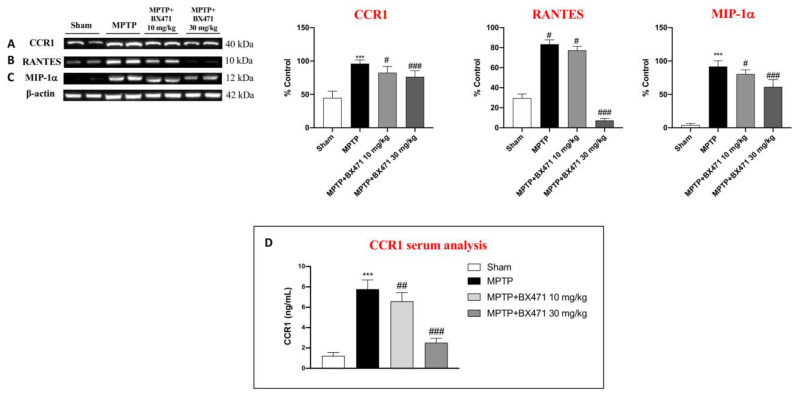
BX471 administration modulated CCR1, RANTES, and MIP-1a. Representative blots of CCR1 (**A**), RANTES (**B**), MIP-1a (**C**). Serum evaluations of CCR1 was also performed (**D**). In every experimental group, the number of mice was *n* = 8. Values are means  ±  SD. One-Way ANOVA test. *** *p*  <  0.001 vs. Sham; # *p*  <  0.05 vs. MPTP; ## *p*  <  0.01 vs. MPTP; ### *p*  <  0.001 vs. MPTP.

The obtained data demonstrated that CCR1, RANTES, and MIP-1a expression was meaningfully upregulated in brain samples after intraperitoneal MPTP injection compared to the Sham mice (Figure 7A–C). Unlike MPTP-injured mice, proteins expression in the brain of BX471-treated mice were drastically downregulated in a dose-dependent manner (Figure 7A–C), confirming the ability of this molecule to block CCR1 activity and the related signaling pathway.

Furthermore, we assessed circulating levels of CCR1 in the serum of mice, taking into account the few data supporting its function as a pathogenic biomarker in PD.

Within this framework, our investigation revealed that MPTP-injected mice exhibited increased serum levels of CCR1 (Figure 7D). BX471 intraperitoneal injections dramatically decreased the circulating levels of CCR1 in the serum, particularly at the higher dosage of 30 mg/kg (Figure 7D).

### 2.8. Block of CCR1 Receptor by BX471 Reduced Mast Cell Chymase and Tryptase Expression after MPTP-Induced Nigrostriatal Degeneration

To further support the previously established relationship between mast cells and CCR1, we used immunofluorescence labeling of mast cells tryptase and chymase.

**Figure 8:**
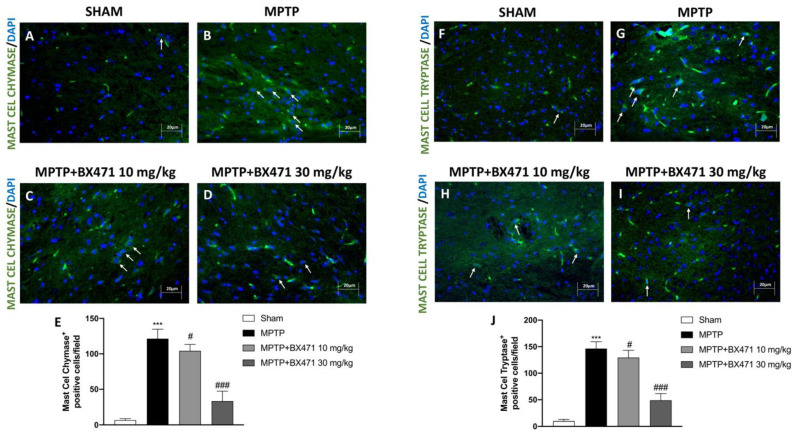
BX471 administration decreased the activity of Mast Cell Chymase and Tryptase. Representative images of immunofluorescence analysis for Mast Cell Chymase and Tryptase markers: Sham (**A** and **F**, respectively), MPTP (**B** and **G**, respectively), MPTP + BX471 10 mg/kg (**C** and **H**, respectively), MPTP + BX471 30 mg/kg (**D** and **I**, respectively). Scores are illustrated (**E**,**J**). In every experimental group, the number of mice was *n* = 8. White arrows indicate positive cells. Figures are shown at 40× magnification. Values are means  ±  SD. One-way ANOVA test. *** *p*  <  0.001 vs. Sham; # *p*  <  0.05 vs. MPTP; ### *p*  <  0.001 vs. MPTP.

Comparing MPTP-injured mice (Figure 8B and Figure 8G, respectively, score Figure 8E and Figure 8J) to the Sham group (Figure 8A and Figure 8F, respectively, score Figure 8E and Figure 8J), a significant increase in positive cells for mast cell chymase and tryptase was detected.

The intraperitoneal administration of BX471, inhibiting CCR1, was shown to be very effective in modulating the expression of mast cell granules constituents (Figure 8C and Figure 8H, respectively, score Figure 8E and Figure 8J), with a stronger statistical significance at a dosage of 30 mg/kg (Figure 8D and Figure 8I, respectively, score Figure 8E and Figure 8J).

### 2.9. Block of CCR1 by BX471 Decreased T Lymphocyte Infiltration into the Midbrain Induced by MPTP Intoxication

The number of CD4^+^ and CD8^+^ antigen-expressing cells, two trustworthy indicators of the T cell activation process, was assessed using immunofluorescence analysis.

**Figure 9:**
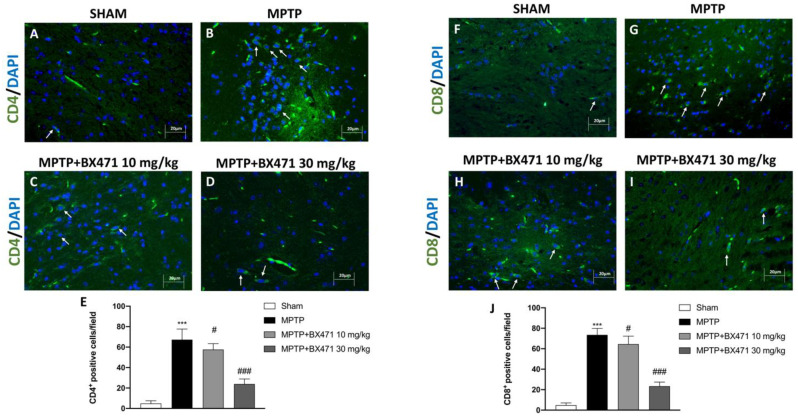
BX471 modulated CD4 and CD8 expression. Representative images of immunofluorescence analysis for CD4 and CD8 markers: Sham (**A** and **F**, respectively), MPTP (**B** and **G**, respectively), MPTP + BX471 10 mg/kg (**C** and **H**, respectively), MPTP + BX471 30 mg/kg (**D** and **I**, respectively). Scores are illustrated (**E**,**J**). In every experimental group, the number of mice was *n* = 8. White arrows indicate positive cells. Figures are shown at 40× magnification. Values are means  ±  SD. One-Way ANOVA test. *** *p*  <  0.001 vs. Sham; # *p*  <  0.05 vs. MPTP; ### *p*  <  0.001 vs. MPTP.

Following the intraperitoneal administration of MPTP, the total number of CD4^+^ and CD8^+^ cells in the brain sections was significantly higher (Figure 9B and Figure 9G, respectively, score Figure 9E and Figure 9J) than in the tissues of the Sham animals (Figure 9A and Figure 9F, respectively, score Figure 9E and Figure 9J).

Blocking of CCR1 exerted by BX471 administration notably shifted immune response significantly reducing the number of CD4 and CD8 T cell surface positive cells (Figure 9C and Figure 9H, respectively, score Figure 9E and Figure 9J), particularly at the maximum dose of 30 mg/kg (Figure 9D and Figure 9I, respectively, score Figure 9E and Figure 9J).

## 3. Discussion

The present study aimed to shed light on the crosstalk between neuroinflammation and altered neuroimmunity in the context of PD, since this interplay largely contribute to dopaminergic neurons death. In particular, we focused our attention on CCR1 chemokine receptor that is present in neurons, microglia, and astrocytes where likely promotes neuroinflammation during the establishment of CNS disorders such as PD, acting as a trigger of several pro-inflammatory signaling pathways like the NF-κB one [24,25,26].

Previous reports had found that the chemokine receptor CCR1 was present in neurons and dystrophic processes in a small sample of AD cases [27], suggesting its overexpression as an early marker for this pathology and also opening new horizons about the role of this receptor in other neurodegenerative diseases including PD.

These hypotheses were further reinforced by last ten years of research on peripheral blood and cerebrospinal fluid from PD patients, which reveals changes in immune cell populations and inflammatory markers that may cause or worsen neuroinflammation and prolong the neurodegenerative process [28]. This has led to the theory that the immune system, in conjunction with intricate gene-by-environment interactions, creates the “perfect storm” that permits the onset and progression of PD [28], where immune-related receptors like CCR1 may have clinical relevance. CCR1 is expressed in many cells and organs including the brain [20]. The mechanism by which CCR1 is inhibited by BX471 has not been established yet, but it is thought that BX471 binds directly to CCR1. BX471, interfering with the signaling cascade related to CCR1 activation, consequently reduces the expression of this receptor [20]. In particular, Vaidehi and colleagues found that Tyr-113 and Tyr-114 on transmembrane domain 3 and Ile-259 on transmembrane 6 contribute significantly to the binding of BX471 to CCR1 [29].

Therefore, we thoroughly examined CCR1’s role in spreading neuroinflammation and altered immune system response in the context of MPTP-induced nigrostriatal degeneration in mice by taking advantage of BX471 antagonism action. In addition, through further analyses performed on mice serum, we also assessed CCR1’s potential utility as a pathological biomarker.

As has been well documented, dopamine circuit impairment is the direct cause of motor dysfunctions including bradykinesia, stiffness, and tremor, all typical features of PD patients [30].

Furthermore, PD patients’ clinical picture is exacerbated by cognitive impairments and emotional deficiencies that persistently impact their well-being and daily activities, overall resulting in a significant decrease in the quality of life [31].

Regarding this, our results clearly demonstrated that the injection of MPTP led to a decrease in motor and non-motor skills in mouse model of PD. Otherwise, by blocking CCR1, BX471 treatment greatly reduced the loss of locomotor agility and enhanced the mice’s emotional state, thus promoting the restoration of both motor and non-motor behavioral functions.

The catalytic conversion of L-tyrosine to l-3,4-dihydroxyphenylalanine (L-DOPA), which is the first and rate-limiting step in the production of catecholamines, is dependent on TH, an enzyme that thus covers an important role in PD pathophysiology [32].

Indeed, as demonstrated by many post-mortem investigations, the change in TH content has the predictive validity to assess the likelihood of catecholamine malfunction and PD development [33].

In accordance, our findings demonstrated a significant reduction of TH content in MPTP-injured mice. On the other hand, BX471 intraperitoneal injection meaningfully raised TH expression in neurons, safeguarding CNS homeostasis against the neuronal damage caused by MPTP

The maintenance of nigrostriatal dopamine balance is critical for the survival of neurons in the midbrain [34]. In this scenario, it has been demonstrated that neurons are capable of altering dopamine clearance in response to physiological demands thanks to DAT action, which regulates dopamine feedback signals [35].

To be more precise, DAT activity causes dopamine to be rapidly taken up by presynucleinaptic neurons from the extracellular environment, and this course is crucial for controlling the strength and duration of dopaminergic transmission [36].

The results of this investigation demonstrated that MPTP significantly reduced DAT expression in the substantia nigra. Rather, BX471-treated mice revealed a noteworthy increase in DAT levels in the midbrain region, confirming the neuroprotective properties of this CCR1 antagonist.

The shift of α-synuclein from its physiological synuclein position to aggregates in degenerating neuronal cell bodies has also been extensively related to PD etiology [37]. It has been shown that α-synuclein undergoes a multi-step process under pathological settings that results in the development of soluble oligomer species, toxic for neurons health [37]. Regarding this, the most common species of the harmful aggregates is α-synuclein that has undergone aberrant phosphorylation, a pathogenic alteration that promotes the development of fibrils and insoluble aggregation [38].

Our findings showed that MPTP-injured animals had higher expressions of both α-synuclein and its phosphorylated form. However, a successful reduction in synucleinopathy bought on by MPTP injection was evidenced following BX471 treatment.

The development of a persistent neuroinflammatory state under pathological settings, such as neurodegenerative illnesses, activated glia and maintained the processes of neuronal degeneration during PD [39]. In this framework, GFAP and IBA-1 expressions were particularly enhanced as a result of the increase in reactive astrocytes and microglia, thus representing trustworthy indicators of brain damage related to neurodegeneration [40].

Herein, after MPTP-induced nigrostriatal degeneration, our data demonstrated a significant alteration in both GFAP and IBA-1 expression. In this study, BX471 treatment reduced reactive astrocytes and microglia by blocking CCR1 activity, as seen by the lowered expression of both markers GFAP and IBA-1.

The inflammatory cascade is a key pathophysiological component in the complex etiopathogenesis of PD, since multiple signaling pathways are activated by inflammation, largely contributing to the poor course of the disease [41].

A crucial component in coordinating the many cellular processes that underlie inflammation is the transcriptional factor NF-κB [42]. The production of many pro-inflammatory mediators, including cytokines, chemokines, and enzymes, which are stimulated by this nuclear factor, leads to the worsening of the illness [42].

In this report, BX471 treatment via CCR1 receptor blockade showed advantageous effects, moderating the NF-κB pathway, proinflammatory enzyme, and cytokine expressions both in the brain and in serum.

Moreover, the CCR1 switching off by BX471 also downregulated the expression of immunoinflammatory ligands such as RANTES and MIP1-α.

These findings proved that CCR1 inhibition helps to reduce the alteration of neuroimmune and neuroinflammatory states brought on by PD.

PD may be exacerbated by immune system dysfunction, according to the recent literature data [43]. These records include evidence of immune dysregulation in experimental models of PD, impaired humoral and cellular immune responses in PD, as well as clinical and genetic associations between autoimmune diseases and PD [43]. In particular, concerning the interplay between CCR1 and immune response, it was demonstrated that this chemokine receptor is largely expressed on the surface of mast cells [44]. Indeed, in in vitro models, co-stimulation of CCR1 results in greater mast cells degranulation [44].

Further, according to Schaller et al. [45] CD4^+^ T cells from CCR1^−/−^ mice produced fewer Th1 and Th2 cytokines like IFN-γ and IL-4 or IL-13, respectively, suggesting that CCR1 may represent a significant T-cell chemokine receptor during inflammatory reactions.

After injecting MPTP, we observed an increased infiltration of mast cells and an uncontrollably activated T cell response in the midbrain. Nonetheless, T cell markers (CD4^+^ and CD8^+^) and mast cell indicators (chymase, tryptase) were considerably downregulated in brain tissues following BX471 administration.

Despite the promising results of this study, some limitations should be taken into consideration. Firstly, preclinical models do not always accurately represent human diseases in a translational manner. Indeed, unlike clinical PD, MPTP injections only causes a prompt neurodegeneration, not fully recapitulating the PD clinical course in humans. Furthermore, patients may have compensatory alterations during the progression of the illness that would not be possible in short-term animal models [46]. It is also important to underline the differences in innate and adaptive immunity between humans and mice. In this regard, since we were able to detect immune cells assessing individual markers using microscopy-based techniques, the application of advanced methods like mass cytometry or flow cytometry will be able to better validate these preliminary results and provide a more robust characterization of the immunomodulatory shift during PD progression.

In addition to the need to carry out more in-depth preclinical studies, these results should also be validated through clinical studies. Currently, the blockade of chemokine signaling through antagonist employment was investigated by Zipp and colleagues in a clinical trial comprising 105 patients suffering from relapsing-remitting multiple sclerosis (RRMS) [47]. Their research sought to clarify if the inhibition of CCR1 could affect immune cell infiltration into the CNS; however, they failed to provide a substantial positive impact [47]. This underlines how it is necessary to investigate even more the possible therapeutic applications of CCR1 antagonists such as BX471, not only testing it with different concentrations or routes of administrations, but also in other different CNS pathologies.

## 4. Materials and Methods

### 4.1. Materials

BX471 was purchased by MedChemExpress (Monmouth Junction, NJ, USA); detailed datasheets are available from the manufacturer’s website. Unless explicitly stated, all materials were purchased from Sigma-Aldrich Company Ltd. (Milan, Italy). The highest quality commercial-grade chemicals were available for the other compounds. In non-pyrogenic saline (0.9% NaCl; Baxter, Liverpool, UK), all stock solutions were made.

### 4.2. Animals

CD1 adult mice (6–8 weeks old, male, 25 to 30 g, Envigo, Italy) were housed in a controlled environment (22 ± 2 °C, 55 ± 15% relative humidity, 12 h light/dark cycle). Standard diet and water were available ad libitum. Before starting the experiment, the animals were housed in a quarantine section and monitored for a week to assess their suitability for the study. Animal care was conducted in accordance with Italian laws protecting animals used in research and other experimental settings. The animal study was performed following Italian regulations on the use of animals (D.M.116192), Directive legislation (EU) (2010/63/EU), and ARRIVE guidelines.

### 4.3. MPTP-Induced Nigrostriatal Degeneration

For the experimental model, at day 0, male CD1 mice received four intraperitoneal injections of MPTP (20 mg/kg; Cayman Chemical Company, Ann Arbor, MI, USA) in saline at 2 h intervals; the total dose for each mouse was 80 mg/kg. The MPTP dose of 80 mg/kg was determined from previous in vivo research [48]. Starting 24 h after the first MPTP injection, animals received intraperitoneal administration of BX471 at doses of 3 mg/kg, 10 mg/kg, and 30 mg/kg. Following the manufacturer’s instruction, BX471 was dissolved in 10% DMSO, 40% PEG300, 5% Tween-80, and 45% saline. Intraperitoneal administration was given once daily until the end of the experiment. The dose and route of administration of BX471 were based on previous in vivo studies [22]. Mice were euthanized 7 days following the injection of MPTP, and their brains were collected for histological and biochemical analyses.

### 4.4. Experimental Groups

The animals were assigned to the following groups through random allocation:

**Group 1: Sham + vehicle:** vehicle solution (saline) was injected intraperitoneally during the first day like for the MPTP protocol. In addition, the vehicle solution of BX471 was intraperitoneally administered once daily for 7 consecutive days (*n*  =  8).

**Group 2: MPTP + vehicle:** MPTP solution was injected intraperitoneally during the first day. In addition, the vehicle solution of BX471 was intraperitoneally administered once daily for 7 consecutive days (*n*  =  8).

**Group 3: MPTP + BX471 3 mg/kg:** MPTP solution was injected intraperitoneally during the first day. In addition, the animals received intraperitoneal administration of BX471 3 mg/kg once daily for 7 consecutive days (*n* = 8).

**Group 4: MPTP + BX471 10 mg/kg:** MPTP solution was injected intraperitoneally during the first day. In addition, the animals received intraperitoneal administration of BX471 10 mg/kg once daily for 7 consecutive days (*n* = 8).

**Group 5: MPTP + BX471 30 mg/kg**: MPTP solution was injected intraperitoneally during the first day. In addition, the animals received intraperitoneal administration of BX471 30 mg/kg once daily for 7 consecutive days (*n* = 8).

### 4.5. Behavioral Testing

Behavioral evaluations of each mouse were conducted seven days after the last MPTP injection. Before the onset of the behavioral tests, the animals were allowed to acclimate for 5 min for 2 days in the behavior room [48].

#### 4.5.1. Pole Test

The Pole Test was conducted to examine movement disorders. The test was performed using a gauzed pole (1 cm in diameter) that was 50 cm high. The animals were placed on top of a vertical pole, directed towards their cages. Under natural conditions, the mice will be oriented downwards along the length of the pole. The test’s criteria were the amount of time the animal took to turn 180 degrees, or the turning time, and the total amount of time the animal took to descend to the ground [48].

#### 4.5.2. Elevated plus Maze (EPM)

Anxiety deficits were evaluated using an elevated plus maze (EPM) system. Two open and two closed arms were part of the apparatus used for this test. All the mice were placed separately in the open arm and allowed to explore for five minutes. Animal behavior indications of its emotional state were identified to be the latency of attendance in both closed arms and the center [48].

### 4.6. Stereological Analysis

Unbiased counting of TH^+^ dopaminergic neurons was applied to the substantia nigra pars compacta (SNpc) region, as previously reported [19]. After being exposed to polyclonal primary antibody mouse anti-Tyrosine Hydroxylase (TH) (1:100; sc-25269; Santa Cruz Biotechnology, Santa Cruz, CA, USA) for an overnight period, each slice was processed. After counterstaining brain sections with cresyl violet for Nissl staining, the sections were coated, and TH+ neurons were counted using a specialized program. The figures are shown by 20× and 40×.

### 4.7. Immunohistochemical Localization of Dopamine Transporter (DAT) and α-Synuclein

Immunohistochemistry (IHC) was conducted as described by Campolo et al. [49] and briefly reported below. The sections underwent an overnight incubation with primary antibodies, namely anti-DAT (1:100; sc-14002; Santa Cruz Biotechnology) and anti-α-synuclein (1:100; sc-7011; Santa Cruz Biotechnology), subsequent to the paraffin being removed using a declining scale of alcohols. Using the VECTASTAIN Universal Quick Kit, Peroxidase, R.T.U. (PK-7800; Vector Laboratories, Burlingame, CA, USA), the sections were then meticulously cleaned with PBS before being probed with the secondary antibody. The reaction was observed using the water-soluble chromogenic substrate 3,3′-Diaminobenzidine (DAB), with Nuclear Fast Red serving as a counterstain. The percentage area of immunoreactivity was examined using a computerized image analysis system, and it was determined by counting the number of positive pixels. The proportion of total tissue area (brown staining) was expressed as a percentage among five random fields at a 40× magnification. For the analysis, a Nikon Eclipse Ci-L microscope was used. Figures are shown at 40× magnification.

### 4.8. Immunofluorescence of Glial Fibrillary Acidic Protein (GFAP), Ionized Calcium-Binding Adapter Molecule-1 (IBA-1), Mast Cell Chymase, Mast Cell Tryptase, CD-4, and CD-8

Immunofluorescence staining of GFAP, IBA1, Mast Cell Chymase, Mast Cell Tryptase, CD4, and CD8 was conducted as previously indicated [50]. Following deparaffinization, the following primary antibodies were incubated overnight on the brain slices.: GFAP (1:100; sc-9065 Santa Cruz Biotechnology, Santa Cruz, CA, USA), IBA-1 (1:100; sc-32725 Santa Cruz Biotechnology, Santa Cruz, CA, USA), Mast Cell Chymase (1:100; sc-59586 Santa Cruz Biotechnology, Santa Cruz, CA, USA), Mast Cell Tryptase (1:100; sc-59587 Santa Cruz Biotechnology, Santa Cruz, CA, USA), CD-4 (1:100; sc-514571 Santa Cruz Biotechnology, Santa Cruz, CA, USA), and CD-8 (1:100; sc-7970 Santa Cruz Biotechnology, Santa Cruz, CA, USA). The secondary fluorescent antibodies Alexa Flour 488 goat anti-mouse (A11001; Molecular Probes, Eugene, OR, USA) or anti-rabbit IgG (A11008; Molecular Probes, Eugene, OR, USA) were incubated for three hours the next day, depending on the primary antibody employed. Subsequently, slices were stained with 4′,6′-diamidino-2-phenylindole (DAPI; Hoechst, Frankfurt, Germany). The histological studies were carried out in a blinded manner, and a fluorescence microscope picture (Nikon Eclipse Ci-L microscope, NIKON CORPORATION, Tokyo, Japan) was used to collect. Pictures are shown at 40× magnification.

### 4.9. Western Blot Analysis

To perform the Western Blot, we employed the previously mentioned methodology [51]. In brief, the brain samples were homogenized in order to separate the cytosolic and nuclear fractions. The Bio-Rad protein assay was then used to quantify the protein concentrations, with bovine serum albumin (BSA) serving as the reference. Equal quantities of protein were loaded into a 10–12% SDS-PAGE gel and transferred to PVDF membranes after the proteins were denatured at 95° for five minutes. Subsequently, the membranes were probed with the specific primary antibodies overnight at 4 °C after being blocked for an hour at room temperature using 5% (*w*/*v*) nonfat dried milk in buffered saline (PM). The primary antibodies tested were: anti-nuclear factor of kappa light polypeptide gene enhancer in B-cell inhibitor, alpha (IĸB-α) (1:500; Santa Cruz Biotechnology, sc-1643, Dallas, TX, USA), anti-induced nitric oxide synucleinthase (iNOS) (1:500; 610432, BD Transduction Laboratories, Franklin Lakes, NJ, USA), anti-cyclooxygenase-2 (COX-2) (1:500; sc-376861, Santa Cruz Biotechnology, Dallas, TX, USA), anti-nuclear factor kappa-light-chain-enhancer of activated B cells (NF-κB) (1:500; sc-8008, Santa Cruz Biotechnology, Dallas, TX, USA), anti-TNF- α (1:500; sc-52746, Santa Cruz Biotechnology, Santa Cruz, CA, USA), anti-IL-1β (1:500; sc-32294, Santa Cruz Biotechnology, Santa Cruz, CA, USA), anti-CCR1 (1:500; PA1-41062, ThermoFisher, Waltham, MA, USA), anti-RANTES (1:500; P230E, ThermoFisher, Waltham, MA, USA), and anti-CCL3 (MIP-1α) (1:500; PA5-46951, ThermoFisher, Waltham, MA, USA) in 1× PBS, 5% *w*/*v* nonfat dried milk, and 0.1% Tween-20 (PMT) at 4 °C overnight. After that, membranes were incubated for one hour at room temperature with a secondary antibody, either anti-mouse or anti-rabbit (1:1000, Jackson ImmunoResearch, West Grove, PA, USA). Additionally, membranes were coated with the antibodies LAMIN A for the nuclear fraction (1:500; sc-518013; Santa Cruz Biotechnology, Dallas, TX, USA) or β-actin for the cytosolic fraction (1:500; sc-47778; Santa Cruz Biotechnology, Dallas, TX, USA). Following the manufacturer’s instructions, signals were provided using the Enhanced Chemiluminescence (ECL) detection system reagent (ThermoFisher, Waltham, MA, USA). Utilizing the Bio-Rad ChemiDocTMXRS+ software(Version 3.0.1), densitometry was used to quantify the relative expression of the protein bands, with the levels of β-actin or LAMIN A/C serving as internal controls.

### 4.10. ELISA Kit

Phospho-α-synuclein (p-α-synuclein) on brain tissue extracts, as well as CCR1, TNF-α, and IL-β on serum, were evaluated using ELISA kits, as previously described [52]. Measurements were acquired using a microplate reader, and analyses were carried out in accordance with the manufacturer’s instructions.

### 4.11. Statistical Analysis

All experimental data in this work are given as mean ± standard error (SD) of N observations, where N is the total number of animals analyzed. Numerical data of the experiment expressed as mean ± SD are provided in the Supplementary File (Table S1). For data analysis, GraphPad version 8.0 (La Jolla, CA, USA) was utilized. For multiple comparisons, a One-Way ANOVA and a Bonferroni post hoc test were employed; a *p*-value of less than 0.05 was the threshold for statistical significance [53].

## 5. Conclusions

Collectively, these findings offer a novel overview of immune-inflammatory reactions in PD, assessing for the first time the complex function of CCR1 as the primary mediator of various signaling pathways. The use of CCR1 antagonists, such as BX471, provided beneficial effects that include improved control of PD symptoms, decreased neuroinflammation and activation of the NFkB pathway, as well as decreased infiltration of immune cells after MPTP-induced nigrostriatal degeneration. Considering these new insights and the increase of CCR1 levels in serum following PD establishment, CCR1 might be a pathogenic marker and a useful target for innovative immunotherapeutic strategies in the perspective of dopaminergic neuron survival. This could constitute an important advancement in the therapeutic strategy for PD patients, likely improving their daily activities and quality of life. Moreover, given that studies are increasingly recognizing the role of immune dysregulation and inflammation driven by chemokines such as CCL18 in the pathogenesis of Gaucher disease, CCR1 modulation could also be relevant for the Gaucher patients developing PD. Nevertheless, future studies are required to fully explore the role of CCR1 in CNS diseases and validate these encouraging findings in well-designed clinical trials.

## Data Availability

All data in this study are included in this published article.

## Supplementary Materials

The following supporting information can be downloaded at: https://www.mdpi.com/article/10.3390/ijms25084337/s1.
