# Supplementary material for: Novel Findings on CCR1 Receptor in CNS Disorders: A Pathogenic Marker Useful in Controlling Neuroimmune and Neuroinflammatory Mechanisms in Parkinson’s Disease
Source: Int J Mol Sci. 2024 Apr 14;25(8):4337. doi: 10.3390/ijms25084337 (PMC11050472; doi:10.3390/ijms25084337)
Supplement: Supplementary file 1 [file ijms-25-04337-s001.zip › ijms-2920757-supplementary.pdf]

	SHAM	MPTP	MPTP+BX471 3 mg/kg	MPTP+BX471 10 mg/kg	MPTP+BX471 30 mg/kg
POLE TEST "Time to turn"	1,00 ± 0,535	5,38 ± 0,518	5,25 ± 0,463	4,50 ± 0,535	2,75 ± 0,463
POLE TEST "Total Time"	6,63 ± 2,39	26,9 ± 1,73	26,6 ± 1,30	23,8 ± 1,67	10,0 ± 2,45
EPM TEST "Time in closed arm"	37,0 ± 13,2	237 ± 28,6	235 ± 20,2	206 ± 10,0	96,3 ± 6,18
EPM TEST "Total Time"	5,88 ± 2,17	22,1 ± 1,81	21,5 ± 1,31	19,5 ± 1,07	11,5 ± 1,60
Nissl Staining TH	409 ± 46,2	169 ± 22,2	178 ± 36,4	238 ± 53,5	377 ± 45,8
IHC DAT	6,13 ± 0,354	1,50 ± 0,535	1,63 ± 0,518	2,38 ± 0,518	4,50 ± 0,535
IHC α-syn	0,625 ±0,518	6,63 ± 0,744	6,38 ± 0,518	5,63 ± 0,744	2,13 ± 0,354
ELISA Kit p-α-syn	4,55 ± 1,03	91,2 ± 4,40	88,2 ± 3,25	83,5 ± 6,55	23,4 ± 5,51
IF GFAP	2,38 ± 0,744	19,1 ± 1,55	/	17,1 ± 0,835	6,88 ± 1,81
IF IBA-1	2,13 ± 0,641	27,6 ± 2,92	/	24,4 ± 2,56	7,38 ± 1,41
WB IkBα	98,0 ± 13,6	10,1 ± 5,51	/	28,6 ± 8,95	116 ± 14,6
WB INOS	15,2 ± 2,94	67,7 ± 11,7	/	49,6 ± 14,0	15,8 ± 3,77
WB COX-2	2,82 ± 2,08	103 ± 8,27	/	65,9 ± 10,5	28,4 ± 9,78
WB NFκB	1,30 ± 0,628	70,1 ± 7,15	/	60,7 ± 7,59	8,49 ± 3,47
WB TNF-α	11,4 ± 6,19	93,1 ± 10,9	/	79,5 ± 8,08	15,5 ± 3,52
WB IL-1β	2,60 ± 1,60	75,9 ± 6,22	/	60,9 ± 9,97	29,2 ± 7,09
ELISA Kit TNF-α	18,8 ± 11,3	640 ± 38,2	/	566 ± 59,7	245 ± 59,4
ELISA Kit IL-1β	23,9 ± 8,97	1087 ± 72,7	/	952 ± 93,8	440 ± 108
WB CCR1	38,5 ± 8,36	94,8 ± 4,35	/	82,6 ± 9,25	43,9 ± 6,98
WB RANTES	29,5 ± 4,22	83,5 ± 4,31	/	77,4 ± 4,01	7,30 ± 2,03
WB MIP-1α	4,07 ± 2,44	91,9 ± 8,56	/	80,6 ± 6,16	61,3 ± 10,7
ELISA Kit CCR1	1,23 ± 0,317	7,77 ± 0,908	/	6,58 ± 0,856	2,52 ± 0,426
IF MAST CELL CHYMASE	1,13 ± 1,36	13,6 ± 1,85	/	11,4 ± 1,69	4,38 ± 1,06
IF MAST CELL TRYPTASE	0,875 ±0,835	15,0 ± 1,20	/	13,4 ± 1,19	3,88 ± 0,835
IF CD4	4,88 ± 2,70	67,3 ± 10,4	/	57,6 ± 5,78	23,9 ± 5,03
IF CD8	4,85 ± 2,17	73,5 ± 6,35	/	64,5 ± 7,76	23,4 ± 4,03

S1. Numerical data of the experiment expressed as mean ± SD
